# Leak Detection and Location of Water Pipes Using Vibration Sensors and Modified ML Prefilter

**DOI:** 10.3390/s17092104

**Published:** 2017-09-13

**Authors:** Jihoon Choi, Joonho Shin, Choonggeun Song, Suyong Han, Doo Il Park

**Affiliations:** 1 School of Electronics and Information Engineering, Korea Aerospace University, Goyang-City, Gyeonggi-do 10540, Korea; choonggeunsong@gmail.com (C.S.); hhssyy444@gmail.com (S.H.); 2 Energy Solution/Business Team, LG CNS Co., Ltd., Yeongdeungpo-gu, Seoul 07320, Korea; joonho@lgcns.com (J.S.); dipark@lgcns.com (D.I.P.)

**Keywords:** leakage detection, vibration sensor, time difference estimation, generalised cross-correlation, maximum-likelihood (ML) prefilter

## Abstract

This paper proposes a new leak detection and location method based on vibration sensors and generalised cross-correlation techniques. Considering the estimation errors of the power spectral densities (PSDs) and the cross-spectral density (CSD), the proposed method employs a modified maximum-likelihood (ML) prefilter with a regularisation factor. We derive a theoretical variance of the time difference estimation error through summation in the discrete-frequency domain, and find the optimal regularisation factor that minimises the theoretical variance in practical water pipe channels. The proposed method is compared with conventional correlation-based techniques via numerical simulations using a water pipe channel model, and it is shown through field measurement that the proposed modified ML prefilter outperforms conventional prefilters for the generalised cross-correlation. In addition, we provide a formula to calculate the leak location using the time difference estimate when different types of pipes are connected.

## 1. Introduction

This paper deals with the leakage detection and location of water pipes which is one of the main concerns in the water management field. It has been reported that the amount of non-revenue water reaches 15.2–35.1% of drinking water supply in United States, 6.5–24.6% in Europe, and 4.3–27.0% in Korea [1,2,3]. These losses can be classified into unbilled public usage, apparent losses including unauthorized consumption and metering inaccuracies, and real losses through overflows at storage tanks and burst leaks in distribution pipelines caused by bad connections, pipe corrosion, and physical damages. It is especially very important to detect the burst leaks in buried water pipes in order to reduce water production costs as well as to protect public safety [1,4,5].

Most of the water pipes are buried underground, making it difficult to find the location of leaks. For this reason, water leakage has usually been detected when water flows out of the ground due to massive leaks in pipes. As a preemptive detection method, an acoustic-based approach is used that an experienced expert scans a suspected area by listening to leak sounds, yet this scheme is extremely time-consuming and the accuracy highly depends on the detection skills of the personnel [6,7]. As a more systematic approach, a supervisory control and data acquisition system (SCADA) has been considered to periodically measure the flow rate or water pressure using permanently installed sensors [8,9]. This technique has evolved into model-based leak detection that compares the measured data taken on the water network with the predicted values from flow models [10,11], and a model falsification method is introduced for efficiently detecting leaks in large-scale water distribution networks [12,13]. These methods identify the areas with significant water leakage, so additional techniques are required to find the exact position of leaks [8,9,12]. In addition, ground penetration radar can be used to locate leaks in water pipes by inspecting the ground surface and detecting voids in the soil caused by leaking water; however, the use of this approach is limited to specific areas because of high costs and high sensitivity to underground metal objects [4,14]. In [15,16], a non-invasive detection method based on the time domain reflectometry (TDR) has been investigated. This approach finds the position of leaks using the change of the reflection coefficient near leakage points. Because the measured reflection coefficient is sensitive to the temperature and material type around the pipe, robust leak detection is not easy.

The most popular approach to water leak detection is to use acoustic/vibration sensors or pressure transducers attached to the surface of a pipe [5,17,18,19,20,21,22,23,24,25,26,27,28,29]. When two sensors are used, the leakage location can be determined by estimating the time difference through correlation of the receive signals. The performance of various fast Fourier transform (FFT)-based algorithms is compared in terms of the time difference estimation accuracy in [18,19,20,21]; the time difference estimation schemes based on spectral transform are further enhanced by the short-time Fourier transform (STFT) [22] and the wavelet transform [23]; and the correlation-based leak detector has been verified via hardware implementation [24]. In addition, a leak detector is proposed to estimate the time difference using an entropy algorithm [25]. When a single sensor is used, the leakage can be detected using the fact that the change of the frequency spectrum is highly dependent on the change of leakage volume [26]; however, it is not easy to employ this method in real environments due to the sensitivity to the change of frequency response. In [27,28,29], leak detection schemes with single sensor have been investigated based on the wavelet transform techniques; however, it is difficult to adopt in practical environments because they require reference vibration signals without leaks or the detection performance is vulnerable to the system noise. Additionally, other authors are investigating the use of the filter diagonalization method [30], the radio frequency identification (RFID)-based leakage monitoring system [31], and magnetic flux leakage detection [32]. However, because of the excessive complexity, the difficulty of installation and management, and the technical immaturity, these techniques are rarely used in specific areas.

In this paper, we focus on the time difference estimation method using two vibration sensors attached to water pipes. When the power spectral densities (PSDs) and the cross-spectral density (CSD) of the receive signals are known, the optimal time difference estimator is given by the generalised cross-correlation (GCC) with the maximum likelihood (ML) prefilter derived in [18]. Practically, the PSDs and the CSD include certain estimation errors, and thus the ML prefilter may perform worse than the smoothed coherence transform (SCOT) and Wiener processors [21]. In order to improve the performance of the ML prefilter with erroneous PSDs and CSD, we propose a modified ML method that exploits a regularisation factor to mitigate the estimation errors of the PSDs and CSD. We derive a theoretical variance of the time difference estimation error in the discrete-frequency domain through modification of the variance model in the continuous frequency domain [18], and then find the optimal regularisation factor that minimises the theoretical variance in practical water pipe channels. The time difference estimation accuracy of the proposed method is assessed through computer simulations under the channel similar to an actual water pipe. By implementing the vibration sensor in hardware and the proposed detection method in software, respectively, we carry out field measurement in buried water pipes deployed for household use to verify the performance of the proposed leak detector. Numerical simulations and field tests show that the proposed modified ML method performs better than existing correlation-based techniques, in terms of minimising the root mean square deviation (RMSD) of the time difference estimation error and maximising the peak-to-average ratio (PAR) of the cross-correlation function.

This paper is organized as follows. In Section 2, we derive a formula to find the leak location using a time difference between two vibration sensors when two different kinds of pipes are connected. In Section 3, conventional time difference estimation methods are introduced with the signal model, and the modified ML prefilter is proposed for GCC based on the theoretical variance of time difference estimation error. The performance of the proposed prefilter is compared with those of existing techniques through computer simulations and field measurement in Section 4 and Section 5, respectively. Finally, the conclusions are provided in Section 6.

## 2. Locating Leaks Using Time Difference

**Figure 1:**
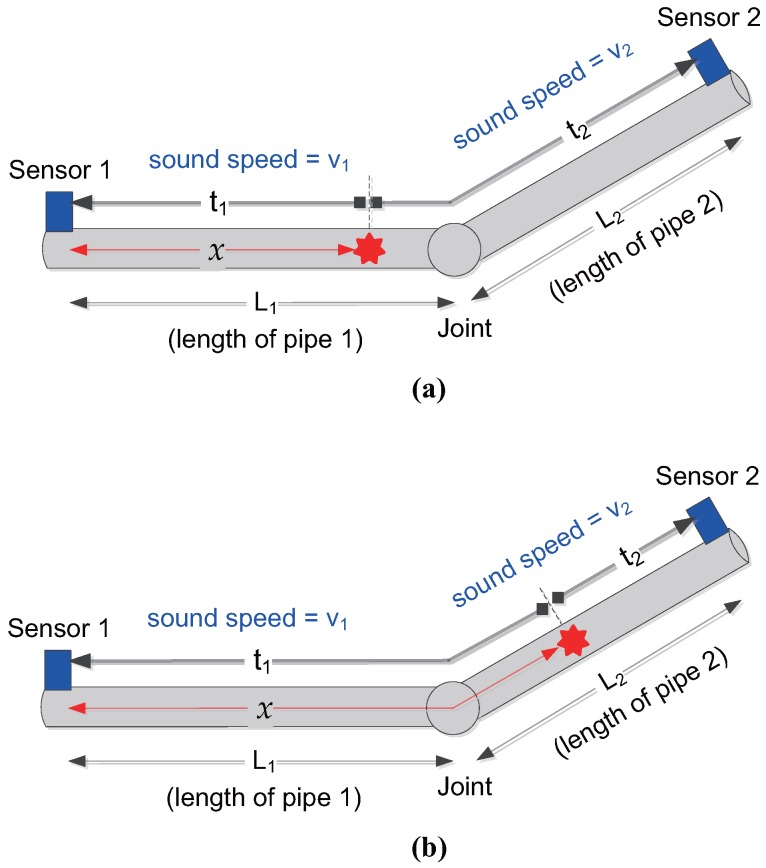
Leak location estimation using the time difference of signals received at sensors, when two different pipes are connected: (**a**) when pipe 1 has a leak; (**b**) when pipe 2 has a leak.

When water is leaked in pipes, internal fluid flows out to generate vibration, and it propagates in both directions from the leak point. As shown in Figure 1, vibration sensors are attached to both ends of the pipes including of a leak point, and thus a difference occurs in the vibration wave reception time due to the difference in distance between the leak point and the sensor. Considering the practical pipeline structure, the material and size of a pipe may be different on both sides of a joint. Define the time difference as

(1) $$\Delta t=t_{1}-t_{2},$$

where $t_{1}$ and $t_{2}$ mean the time for the vibration wave to reach sensors 1 and 2 from the leak point, respectively. The time difference Δt can be estimated by correlating the receive signals of sensors 1 and 2. For the time being, it is assumed that Δt is known, and the estimation of Δt is considered in the following section. Suppose that the pipe 1 has a leak as shown in Figure 1a, i.e., $0\leq x\leq L_{1}$. Then, $t_{1}$ and $t_{2}$ are given by

(2) $$\begin{array}{ccc} t_{1} & = & \frac{x}{v_{1}}, \end{array}$$

(3) $$\begin{array}{ccc} t_{2} & = & \frac{L_{1}-x}{v_{1}}+\frac{L_{2}}{v_{2}}, \end{array}$$

where *x* is the distance between the sensor 1 and the leak point; and $v_{1}$ and $v_{2}$ denote the sound speed in pipes 1 and 2, respectively. From Equations (2) and (3), the time difference is expressed as

(4) $$\Delta t=\frac{2x-L_{1}}{v_{1}}-\frac{L_{2}}{v_{2}}.$$

By rearranging Equation (4) with respect to *x*, we have

(5) $$x=\frac{1}{2}\left(L_{1}+L_{2}\frac{v_{1}}{v_{2}}+v_{1}\Delta t\right),\;$$

where $\Delta t\leq \frac{L_{1}}{v_{1}}-\frac{L_{2}}{v_{2}}$. Similarly, when pipe 2 has a leak as shown in Figure 1b, the time difference is expressed as

(6) $$\Delta t=\frac{L_{1}}{v_{1}}+\frac{2x-2L_{1}-L_{2}}{v_{2}},$$

and the distance between leak point and the sensor 1 is given by

(7) $$x=L_{1}+\frac{1}{2}\left(L_{2}+v_{2}\Delta t-L_{1}\frac{v_{2}}{v_{1}}\right),\;$$

where $\Delta t>\frac{L_{1}}{v_{1}}-\frac{L_{2}}{v_{2}}$. In summary, the leak location from the sensor 1 can be computed as

(8) $$x=\left\{\begin{array}{cc} \frac{1}{2}\left(L_{1}+L_{2}\frac{v_{1}}{v_{2}}\right)+\frac{v_{1}}{2}\Delta t, & \mathrm{for}\;\Delta t_{min}\leq \Delta t\leq \frac{L_{1}}{v_{1}}-\frac{L_{2}}{v_{2}},\\ L_{1}\left(1-\frac{v_{2}}{2v_{1}}\right)+\frac{1}{2}L_{2}+\frac{v_{2}}{2}\Delta t, & \mathrm{for}\;\frac{L_{1}}{v_{1}}-\frac{L_{2}}{v_{2}}<\Delta t\leq \Delta t_{max}, \end{array}\right.$$

where $\Delta t_{max}=\frac{L_{1}}{v_{1}}+\frac{L_{2}}{v_{2}}$ and $\Delta t_{min}=-\Delta t_{max}$.

The speed of sound in a water-filled pipe depends on the properties of a pipe wall that the vibration wave is traveling through. In [33], the velocity of a fluid-dominated wave is expressed as

(9) $$v=v_{f}{\left(1+\frac{\left(2B_{f}/a\right)}{\left(Eh/a^{2}\right)-\rho h\omega^{2}}\right)}^{-\frac{1}{2}},$$

where $B_{f}$ is the bulk modulus of the contained medium; *a*, *h*, and *E* are the inner radius, the thickness, and the elastic modulus of the pipe, respectively; ρ is the density of the surrounding medium; ω is the angular velocity; and $v_{f}$ is the free-field internal fluid velocity [34], given by

(10) $$v_{f}=\sqrt{\frac{K_{s}}{\rho }},\;$$

where $K_{s}$ is a stiffness coefficient of the material. We use the database of the sound speed created for various types of pipes by theoretical calculations and experimental corrections provided by [35], and Table 1 presents a part of the database. Practically, the sound speed varies considerably according to the geometry and material type of the pipe. If a pipe is composed of mixed media, it is difficult to calculate the sound speed. Thus, we only consider a single material pipe.

## 3. Time Difference Estimation

### 3.1. Conventional Time Difference Estimation Methods

In Figure 1, define $x_{1}\left(n\right)$ and $x_{2}\left(n\right)$ as the sampled receive signals of sensors 1 and 2 at time $t=nT_{s}$, respectively, where $T_{s}$ is the sampling period. When there is a leak between sensors 1 and 2, $x_{1}\left(n\right)$ and $x_{2}\left(n\right)$ are given by

(11) $$\begin{array}{ccc} x_{1}\left(n\right) & = & h_{1}\left(n\right)⊗s\left(n\right)+w_{1}\left(n\right), \end{array}$$

(12) $$\begin{array}{ccc} x_{2}\left(n\right) & = & h_{2}\left(n\right)⊗s\left(n+d\right)+w_{2}\left(n\right), \end{array}$$

where s(n) is the vibration wave generated from the leak point; *d* is the time difference in samples between received signals at sensors 1 and 2 that satisfies $\Delta t=dT_{s}$; and ⊗ means the discrete-time convolution operator. For i∈{1,2}, $h_{i}\left(n\right)$ is the discrete-time channel impulse response between the leak point and the sensor *i*, and $w_{i}\left(n\right)$ is a stationary random process representing the background noise at the sensor *i*. It is assumed that $x_{1}\left(n\right)$ and $x_{2}\left(n\right)$ are two stationary random signals uncorrelated with $w_{i}\left(n\right)$, and also assumed that $w_{1}\left(n\right)$ and $w_{2}\left(n\right)$ are uncorrelated. The time difference can be estimated using the cross-correlation of $x_{1}\left(n\right)$ and $x_{2}\left(n\right)$ defined as

(13) $$R_{x_{1}x_{2}}\left(\tau \right)=E\left[x_{1}\left(n\right)x_{2}^{*}\left(n-\tau \right)\right],$$

where τ is the time delay of $x_{2}\left(n\right)$, E[·] denotes the expectation operator, and ${\left(\cdot \right)}^{*}$ means the complex conjugate.

The cross-correlation function $r_{x_{1}x_{2}}\left(\tau \right)$ can be computed in the time domain by directly correlating $x_{1}\left(n\right)$ and $x_{2}\left(n\right)$ because $R_{x_{1}x_{2}}\left(\tau \right)$ has the peak value at τ=d. This approach, however, is very sensitive to the non-leak vibration noises, and requires high computational load when the sample size is large. For these reasons, the time difference estimation method in the frequency domain is widely used based on the relationship between the cross-correlation function and the CSD [18,19,20,21,22,23,24]. Let us define ${\mathrm{DFT}}_{N}\left[x\left(n\right)\right]$ as the *N*-point discrete Fourier transform (DFT) of a sequence x(n). Then, the CSD for (13) is written as

(14) $$\begin{array}{cc} S_{x_{1}x_{2}}\left(k\right) & =E[X_{1}\left(k\right)X_{2}^{*}\left(k\right)]\\ & =H_{1}\left(k\right)H_{2}^{*}\left(k\right)S_{s}\left(k\right)e^{-j\frac{2\pi kd}{N}}, \end{array}$$

where $X_{i}\left(k\right)\;=\;{\mathrm{DFT}}_{N}\left[x_{i}\left(n\right)\right]$ and $H_{i}\left(k\right)\;=\;{\mathrm{DFT}}_{N}\left[h_{i}\left(n\right)\right]$ for i∈{1,2}; $S_{s}\left(k\right)$ is the PSD of s(k); and $-\frac{N}{2}\;\leq \;k\;\leq \;\frac{N}{2}-1$. By computing $S_{x_{1}x_{2}}\left(k\right)$ and taking its inverse DFT (IDFT), the cross-correlation function can be simply computed when the sample size is large.

**Figure 2:**
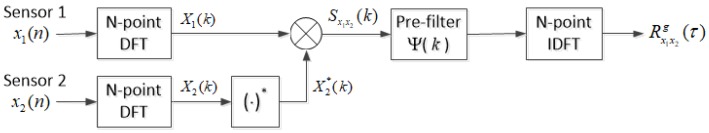
Block diagram to compute $R_{x_{1}x_{2}}^{g}\left(\tau \right)$ using the generalised cross-correlation method.

To further enhance the frequency domain estimation method by sharpening the peak in the cross-correlation function, prefiltering is performed before the IDFT. In this case, the cross-correlation function is computed using the GCC method [18], defined as

(15) $$R_{x_{1}x_{2}}^{g}\left(\tau \right)={\mathrm{IDFT}}_{N}\left[\Psi \left(k\right)S_{x_{1}x_{2}}\left(k\right)\right],$$

where ${\mathrm{IDFT}}_{N}\left[x\left(k\right)\right]$ means the *N*-point IDFT of a sequence x(k) and Ψ(k) is a frequency domain prefilter. Figure 2 describes the procedure to compute the cross-correlation function in the GCC method, and Table 2 presents the prefilters for the GCC proposed in [18,19,20], where $S_{w_{i}}\left(k\right)$ and $S_{x_{i}}\left(k\right)$ are the PSDs of $w_{i}\left(n\right)$ and $x_{i}\left(n\right)$ for i∈{1,2}, respectively; and γ(k) is the coherence function between $x_{1}\left(n\right)$ and $x_{2}\left(n\right)$ defined as

(16) $$\gamma \left(k\right)=\frac{S_{x_{1}x_{2}}\left(k\right)}{\sqrt{S_{x_{1}}\left(k\right)S_{x_{2}}\left(k\right)}}.$$

In the Wiener and ML prefilters, the background noises $w_{1}\left(n\right)$ and $w_{2}\left(n\right)$ are assumed to be zero-mean Gaussian random variables with variances $\sigma_{1}^{2}$ and $\sigma_{2}^{2}$, respectively, to make the signal models (11) and (12) mathematically tractable. As pointed out in [12,13], $E[w_{i}\left(n\right)]$ is not zero due to the sensor bias, and the modeling error included in $w_{i}\left(n\right)$ may not be expressed as a Gaussian distribution. The effect of the bias in $w_{i}\left(n\right)$ can be removed by sensor calibration or subtracting the mean value from $x_{i}\left(n\right)$. In addition, for a large *N*, $W_{i}\left(k\right)={\mathrm{DFT}}_{N}\left[w_{i}\left(k\right)\right]$ tends to be Gaussian by the central limit theorem, even if $w_{i}\left(n\right)$ is not a Gaussian random variable. Hence, the assumption that $w_{1}\left(n\right)$ and $w_{2}\left(n\right)$ are Gaussian is not a strong requirement ([36], Ch.3A).

The Roth prefilter proposed by Peter R. Roth normalises the CSD by $S_{x_{1}}\left(k\right)$, while the smoothed coherence transform (SCOT) prefilter normalises the CSD by $\sqrt{S_{x_{1}}\left(k\right)S_{x_{2}}\left(k\right)}$. The Roth and SCOT prefilters can be used only in the high signal-to-noise ratio (SNR) region because the peak of the cross-correlation function is spread out after prefiltering. To avoid the peak spreading, the phase transform (PHAT) normalises the CSD by $|S_{x_{1}x_{2}}\left(k\right)|$ so that the prefiltered CSD has the unit amplitude for all *k*. However, the peak estimation performance is degraded due to the noise enhancement in the frequency band with low SNR. The Eckart prefilter maximises the ratio of the mean correlator output power to the noise variance of the correlator output, and thus it suppresses the frequency band with low SNR. This method is difficult to use in a practical system because it is not easy to estimate the noise PSDs $S_{w_{1}}\left(k\right)$ and $S_{w_{2}}\left(k\right)$ from the receive signals with leaks [18,20,21]. The ML prefilter weights the CSD according to the SNR in each frequency band to minimise the variance of the time difference estimate, yet it may critically overweight the CSD in the frequency band where $|\gamma_{x_{1}x_{2}}{\left(k\right)|}^{2}$ is close to one [18]. The Wiener prefilter multiplies the CSD by the weight $|\gamma_{x_{1}x_{2}}{\left(k\right)|}^{2}$, so that it mitigates the overemphasising problem at certain frequencies [19].

### 3.2. Proposed Time Difference Estimation Scheme Using the Modified ML Method

For practical implementation of the prefilter, it is required to estimate the PSDs $S_{x_{1}}\left(k\right)$ and $S_{x_{2}}\left(k\right)$ as well as the CSD $S_{x_{1}x_{2}}\left(k\right)$. Additionally, the Eckart prefilter necessitates the estimation of the noise PSDs $S_{w_{1}}\left(k\right)$ and $S_{w_{2}}\left(k\right)$. Suppose that we have long sequences $x_{1}\left(n\right)$ and $x_{2}\left(n\right)$ measured at sensors 1 and 2. Then, the PSDs and CSD of $x_{1}\left(n\right)$ and $x_{2}\left(n\right)$ can be estimated by the non-overlapped Welch’s method [37] as follows:

(17) $$\begin{array}{ccc} {\hat{S}}_{x_{i}}\left(k\right) & = & \frac{1}{M}\sum_{m=1}^{M}{\left|X_{i}^{\left(m\right)}\left(k\right)\right|}^{2}, \end{array}$$

(18) $$\begin{array}{ccc} {\hat{S}}_{x_{1}x_{2}}\left(k\right) & = & \frac{1}{M}\sum_{m=1}^{M}X_{1}^{\left(m\right)}\left(k\right){\left(X_{2}^{\left(m\right)}\left(k\right)\right)}^{*}, \end{array}$$

where $X_{i}^{\left(m\right)}\left(k\right)={\mathrm{DFT}}_{N}\left[x_{i}\left(\frac{mN}{2}\right),x_{i}\left(\frac{mN}{2}+1\right),\cdots ,x_{i}\left(\frac{(m+1)N}{2}-1\right)\right]$; *M* is the number of blocks; and i∈{1,2}. Using the power spectrum estimates in Equation (18), the ML prefilter is expressed as

(19) $$\Psi \left(k\right)=\frac{|\hat{\gamma }{\left(k\right)|}^{2}}{1-|\hat{\gamma }{\left(k\right)|}^{2}}\frac{1}{|{\hat{S}}_{x_{1}x_{2}}\left(k\right)|},$$

where $\hat{\gamma }\left(k\right)$ is the estimated coherence function, given by

(20) $$\hat{\gamma }\left(k\right)=\frac{{\hat{S}}_{x_{1}x_{2}}\left(k\right)}{\sqrt{{\hat{S}}_{x_{1}}\left(k\right){\hat{S}}_{x_{2}}\left(k\right)}}.$$

Note that $0\;\leq \;|\hat{\gamma }\left(k\right)|\;\leq \;1$. When a GCC technique is used to estimate the time difference, the error variance has been derived using the integration of a weighted CSD function in the frequency domain [18]. In the discrete-frequency domain, the error variance for an arbitrary prefilter Ψ(k) is obtained through the summation of a weighted discrete CSD function as follows:

(21) $$\mathrm{var}\left(\hat{d}\right)=\frac{N^{2}}{{\left(2\pi \right)}^{2}}\frac{\sum_{k=-N/2}^{N/2-1}k^{2}\Psi^{2}{\left(k\right)[1-|\gamma \left(k\right)|}^{2}]S_{x_{1}}\left(k\right)S_{x_{2}}\left(k\right)}{{\left[\sum_{k=-N/2}^{N/2-1}k^{2}\Psi \left(k\right)\left|{\hat{S}}_{x_{1}x_{2}}\left(k\right)\right|\right]}^{2}}.$$

To derive the error variance of the ML prefilter using the estimated PSDs and CSD, we substitute Equation (19) into Equation (21). In addition, by approximating that $|S_{x_{1}x_{2}}\left(k\right)|\;≅$ $\;|{\hat{S}}_{x_{1}x_{2}}\left(k\right)|$, we get

(22) $$\begin{array}{cc} \mathrm{var}(\hat{d}) & =\frac{N^{2}}{{\left(2\pi \right)}^{2}}\frac{\sum_{k=-N/2}^{N/2-1}k^{2}{\hat{\beta }}_{0}{\left(k\right)[1-|\gamma \left(k\right)|}^{2}]\frac{S_{x_{1}}\left(k\right)S_{x_{2}}\left(k\right)}{|{\hat{S}}_{x_{1}x_{2}}{\left(k\right)|}^{2}}}{{\left[\sum_{k=-N/2}^{N/2-1}k^{2}{\hat{\beta }}_{0}^{2}\left(k\right)\right]}^{2}}\\ & ≅\frac{N^{2}}{{\left(2\pi \right)}^{2}}\frac{\sum_{k=-N/2}^{N/2-1}k^{2}{\hat{\beta }}_{0}^{2}\left(k\right)\frac{1}{\beta_{0}\left(k\right)}}{{\left[\sum_{k=-N/2}^{N/2-1}k^{2}{\hat{\beta }}_{0}\left(k\right)\right]}^{2}}, \end{array}$$

where $\beta_{0}\left(k\right)={\left|\gamma \left(k\right)\right|}^{2}{/(1-|\gamma \left(k\right)|}^{2})$, and ${\hat{\beta }}_{0}\left(k\right)$ is the estimate of $\beta_{0}\left(k\right)$ given by

(23) $${\hat{\beta }}_{0}\left(k\right)=|\hat{\gamma }{\left(k\right)|}^{2}/(1-|\hat{\gamma }\left(k\right){|}^{2}).$$

It can be easily shown that $\mathrm{var}(\hat{d})$ is minimised when ${\hat{\beta }}_{0}\left(k\right)=\beta_{0}\left(k\right)$ for all *k*. Practically, ${\hat{\beta }}_{0}\left(k\right)$ is highly dependent on the estimation error of γ(k). Especially, when $|\hat{\gamma }\left(k\right)|$ is close to one, ${\hat{\beta }}_{0}\left(k\right)$ is very sensitive to the variation of $\hat{\gamma }\left(k\right)$, thereby resulting in a rapid increase of $\mathrm{var}(\hat{d})$.

In an attempt to mitigate the influence of coherence estimation error, we propose a new modified ML prefilter that employs a regularisation factor when computing ${\hat{\beta }}_{0}\left(k\right)$, and thus it is more robust to the coherence estimation error than the conventional ML prefilter. The proposed modified ML prefilter is expressed as

(24) $$\Psi \left(k\right)=\hat{\beta }\left(k\right)\frac{1}{|{\hat{S}}_{x_{1}x_{2}}\left(k\right)|},$$

where $\hat{\beta }\left(k\right)$ is defined as

(25) $$\hat{\beta }\left(k\right)=\frac{|\hat{\gamma }{\left(k\right)|}^{2}}{1-|\hat{\gamma }{\left(k\right)|}^{2}+\alpha }.$$

Here, 0<α≤1 is the regularisation factor. Since the denominator of Equation (25) is equal to or greater than α, the estimation error included in $\hat{\gamma }\left(k\right)$ gives less impact on the change of $\hat{\beta }\left(k\right)$ compared to the conventional ML prefilter. The proposed modified ML prefilter (24) is equal to the ML prefilter when α=0, while it is equal to the Wiener prefilter when $\alpha =|\hat{\gamma }{\left(k\right)|}^{2}$. By replacing ${\hat{\beta }}_{0}\left(k\right)$ in Equation (22) with $\hat{\beta }\left(k\right)$ in Equation (25), the error variance for the modified ML method is denoted as

(26) $$\mathrm{var}\left(\hat{d}\right)=\frac{N^{2}}{{\left(2\pi \right)}^{2}}\frac{\sum_{k=-N/2}^{N/2-1}k^{2}{\hat{\beta }}^{2}\left(k\right)\frac{1}{\beta_{0}\left(k\right)}}{{\left[\sum_{k=-N/2}^{N/2-1}k^{2}\hat{\beta }\left(k\right)\right]}^{2}}.$$

As the regularization factor α increases, $\hat{\beta }\left(k\right)$ becomes more robust to the estimation error of γ(k); however, the bias of $\hat{\beta }\left(k\right)$ increases. In addition, the optimal α minimising $\mathrm{var}(\hat{d})$ is varied according to SNR values of receive signals and statistical characteristics of γ(k). Therefore, we carefully design α considering the variance of time difference error in numerical simulations and field measurements. The specific design method for α is described in Section 4 and Section 5.

## 4. Simulation Results

### 4.1. Channel Model and Optimisation of Regularisation Factor

To perform computer simulations, we construct a channel model for vibration signals in buried water pipes based on the field measurement data. Details of field measurements are explained in Section 5. We define a channel model for propagation of vibration signals in a water pipe as below:

(27) $$H_{i}\left(z\right)={\left(\frac{d_{i}}{d_{0}}\right)}^{-m}\frac{1}{1+c_{1}z^{-K}}\frac{{\left(1-c_{2}^{2}\right)}^{1/2}}{1-c_{2}z^{-1}}+E_{i}\left(z\right),$$

where $d_{0}=1$ m is the reference distance; $d_{i}$ is the distance between the leak point and the sensor *i*; *m* is the path loss exponent; $-1\leq c_{1}\leq 1$ and *K* are the coefficient and order of a comb filter; $0<c_{2}<1$ is the coefficient of a first-order infinite impulse response (IIR) filter; and $E_{i}\left(z\right)$ is an error term describing the channel variation. It is assumed that, for various ω, $E_{i}\left(e^{j\omega }\right)$ is an independent and identically distributed (i.i.d.) Gaussian random variable with zero mean and variance $\sigma_{h}^{2}$ and that $E_{1}\left(e^{j\omega }\right)$ and $E_{2}\left(e^{j\omega }\right)$ are independent. In Equation (27), $1/(1+c_{1}z^{-K})$ is the inverse comb filter to represent a sharp increase in the frequency response at harmonics of the natural frequency, and the first-order IIR filter describes the lowpass characteristics of vibration signals.

**Figure 3:**
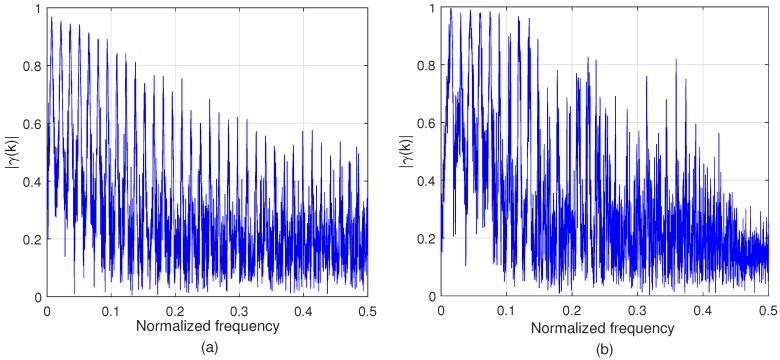
Coherence γ(k) when N=4096, and M=30: (**a**) signals generated by the channel model (27); (**b**) measured signals in the field test for Location 3 and sensors 1 and 3 (L3S13).

Figure 3 compares the coherence function of the signals generated by the channel model (27) and that of the measured signals in the field test, when N=4096 and M=30. For the channel model, we used $d_{1}=20$ m, $d_{2}=30$ m, m=2, $c_{1}=0.7$, K=69, $c_{2}=0.7$, and $\sigma_{h}^{2}=0.02$. In the field measurement, the coherence is obtained by averaging three trials of measurement data for Location 3 and sensors 1 and 3 (L3S13). The two coherence values are very similar except that the normalised frequency is around 0.5, and this shows the validity of the proposed channel model.

**Figure 4:**
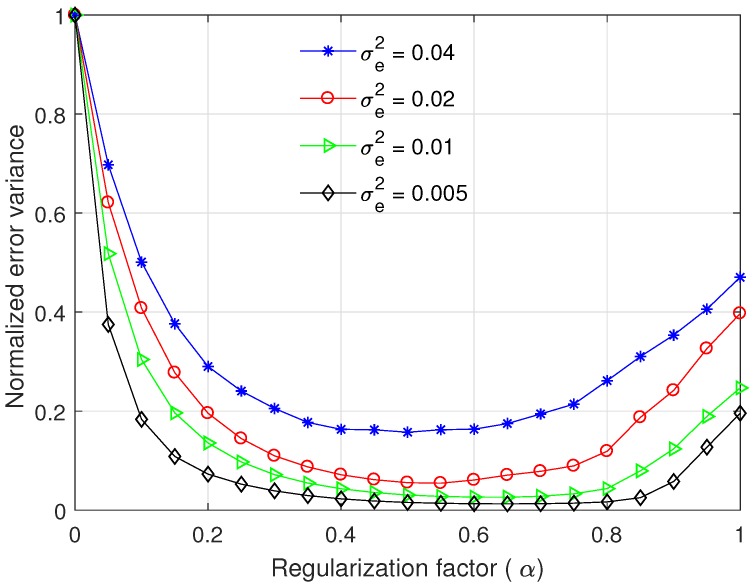
Normalised variance of time difference error as a function of α, when N=4096, and M=30.

Using the channel model (27), we find the optimal regularisation factor α for the proposed modified ML prefilter. In the simulation, the coherence γ(k) is generated using the same parameters as in Figure 3, and the coherence estimate $\hat{\gamma }\left(k\right)$ is denoted as

(28) $$\hat{\gamma }\left(k\right)=\gamma \left(k\right)+\gamma_{e}\left(k\right),$$

where $\gamma_{e}\left(k\right)$ is the estimation error of γ(k) that is assumed to be a zero-mean Gaussian random variable with variance $\sigma_{e}^{2}$. Figure 4 presents the normalised variance of time difference estimation error, which is defined as

(29) $${\mathrm{var}}_{N}\left(\hat{d};\alpha \right)=\frac{\mathrm{var}(\hat{d};\alpha )}{\mathrm{var}(\hat{d};\alpha \;=\;0)},$$

where $\mathrm{var}(\hat{d};\alpha )$ is computed by Equations (25) and (26). The normalised error variance monotonically decreases as the regularisation factor α increases to 0.5, whereas it grows with increment of α when α>0.6. Thus, the normalised error variance is minimised when 0.5≤α≤0.6, and the optimal α is slightly different depending on $\sigma_{e}^{2}$. As $\sigma_{e}^{2}$ decreases, the minimum of normalised error variance decreases, i.e., the performance gain of the modified ML prefilter increases compared to the conventional ML prefilter with α=0.

As mentioned in [12,13], $\gamma_{e}\left(k\right)$ may have a non-Gaussian distribution with a bias in a practical system. However, to the authors’ best knowledge, the distribution of $\gamma_{e}\left(k\right)$ in practical water pipes are not available in the literature, and thus $\gamma_{e}\left(k\right)$ is assumed to be zero-mean Gaussian in the simulation. Since $\mathrm{var}(\hat{d})$ is a function of $\hat{\gamma }\left(k\right)$, the normalised error variance curve in Figure 4 is varied according to the distribution of $\gamma_{e}\left(k\right)$ and the optimal α also can be changed. For more practical design, we compute the normalised error variance curve and the corresponding optimal α using $\hat{\gamma }\left(k\right)$ measured at real test sites in Section 5.2.

### 4.2. Performance Comparison

In this subsection, we compare the performance of the proposed modified ML method with those of existing prefilters. As mentioned in [18,21], the PHAT prefilter performs better than the Roth and SCOT prefilters with the peak spreading problem. In addition, as explained in Section 3, the Eckart prefilter requires the noise PSDs $S_{w_{1}}\left(k\right)$ and $S_{w_{2}}\left(k\right)$, which are difficult to estimate in a practical system. For these reasons, we consider the PHAT, Wiener, and ML prefilters for comparison with the proposed method. To compare the performance of various prefilters, we utilize the RMSD of the time difference estimate given by

(30) $$\mathrm{RMSD}\left(\Delta \hat{t}\right)=T_{s}\sqrt{E[|\hat{d}-d{|}^{2}]}.$$

Note that $\mathrm{RMSD}\left(\Delta \hat{t}\right)=T_{s}\sqrt{\mathrm{var}(\hat{d})}$ when $\hat{d}$ is unbiased. In addition, we employ the PAR to describe the relative magnitude of the maximum peak compared to the average of the cross-correlation function. Specifically, when the maximum peak appears at $\tau =\hat{d}$, the PAR is defined as

(31) $$\mathrm{PAR}\left(\hat{d}\right)=\frac{|R_{x_{1}x_{2}}^{g}\left(\hat{d}\right){|}^{2}}{\frac{1}{N}\sum_{\tau =-N/2}^{N/2-1}{\left|R_{x_{1}x_{2}}^{g}\left(\tau \right)\right|}^{2}},$$

where the cross-correlation function $R_{x_{1}x_{2}}^{g}\left(\tau \right)$ is given by Equation (15).

**Figure 5:**
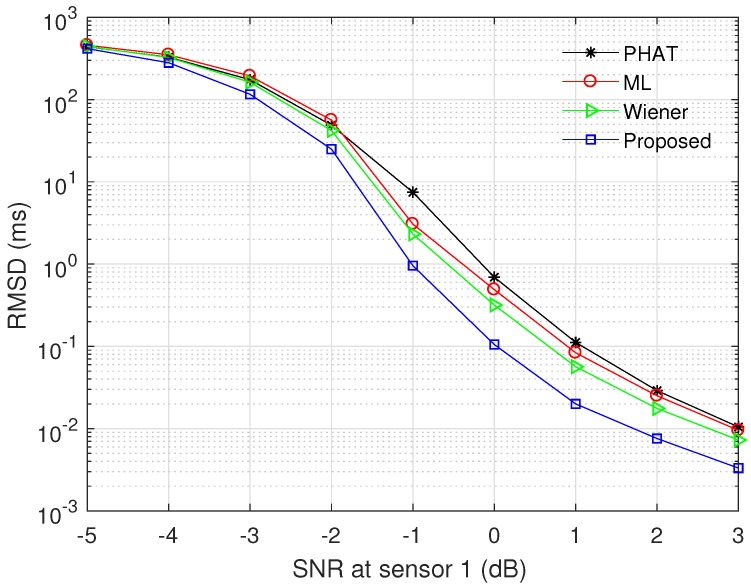
Root mean square deviation (RMSD) of time difference error for various prefilters, when α=0.5, N=4096, and M=30.

**Figure 6:**
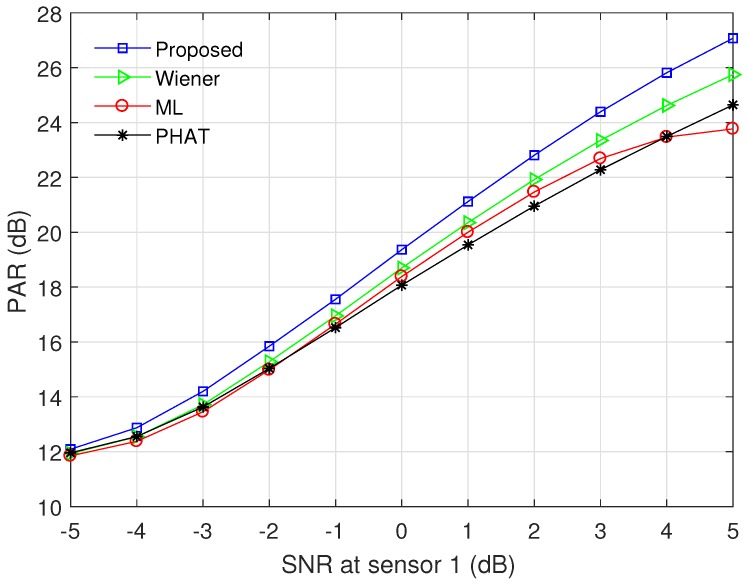
Peak-to-average ratio (PAR) of the time domain cross-correlation function for various prefilters, when α=0.5, N=4096, and M=30.

Figure 5 and Figure 6 show the RMSD and the PAR for various prefilters, respectively, when the channel model (27) is used, N=4096, and M=30. α was set to 0.5 for the proposed modified ML prefilter; $\sigma_{h}^{2}$ was determined by the SNR at sensor 1; *d* was randomly generated in the range between −200 and 200; and other parameters were the same as in Figure 4. All curves were obtained by averaging the simulation results over $10^{4}$ channel realizations. The RMSD decreases with increment of SNR, and the proposed method outperforms the conventional PHAT, ML, and Wiener prefilters in the whole SNR region. The RMSD value of the PHAT prefilter approaches those of the ML and Wiener prefilters as the SNR increases, because the PHAT prefilter has good performance in high SNR region [18,21]. In Figure 6, the proposed modified ML method presents the highest PAR among all prefilters for entire SNR region, i.e., the proposed prefilter can detect the leakage point more reliably than other techniques. The PAR of the ML prefilter is saturated when SNR ≥4 dB because the estimation error of γ(k) influences both the numerator and denominator of Equation (23).

## 5. Field Measurement

### 5.1. System Setup for Field Testing

**Figure 7:**
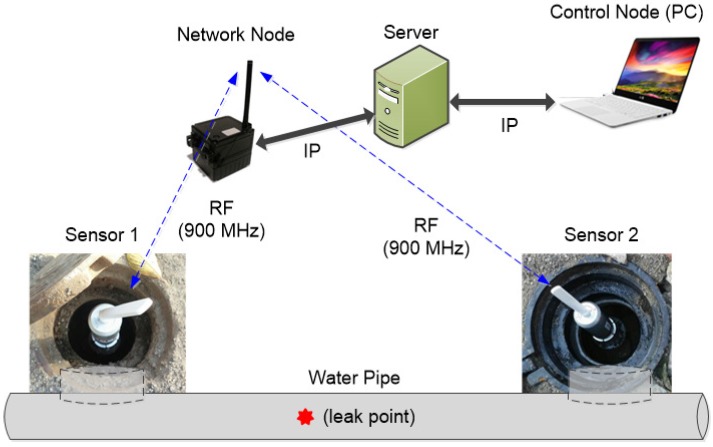
Leak detection system for field measurements.

Figure 7 shows the leak detection system for field measurements. We developed a web-based leakage detection system including our own sensor composed of a vibration transducer, a microprocessor, and a communication module. Once the control node sends the *start logging* command to the sensors via the network node, the sensors record the vibration signals for 30 s and transmit the sampled data to the server through the wireless communication link and the commercial internet protocol (IP) network. Prior to data logging, the network node broadcasts a sync message, and the local clock of the sensor is synchronized using the message to avoid the time difference estimation error caused by the time offset between sensors. The measured data stored in the server can be extracted for leakage detection at the control node. At the sensor, the sampling frequency was 4 kHz ($T_{s}=0.25$ ms) and each sample is represented as 12 bits. The wireless link between the sensor and the network node supports 40 kbps of data rate using binary phase shift-keying (BPSK) modulation.

**Figure 8:**
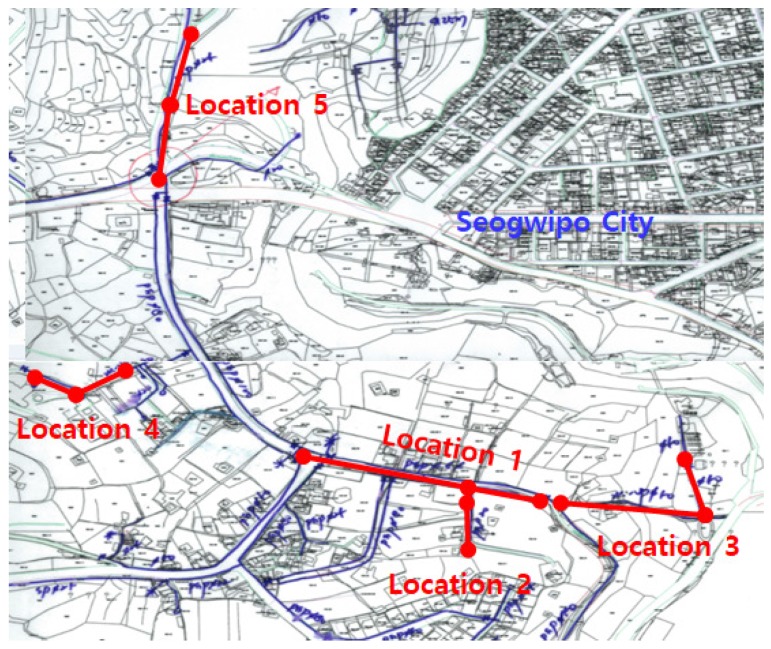
Locations for field measurements.

**Figure 9:**
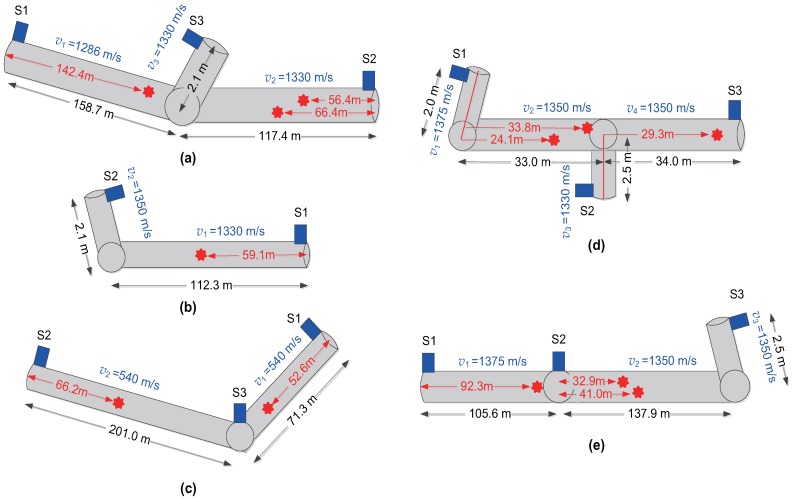
Pipe layout used in field measurements where S1, S2, and S3 means Sensors 1, 2, and 3; and red spots denote leak points: (**a**) Location 1; (**b**) Location 2; (**c**) Location 3; (**d**) Location 4; (**e**) Location 5.

Field measurements were carried out in Seogwipo City, Korea, in order to verify the leak detection performance of the proposed method. To determine specific test areas, we utilized the leak rate information in [3] and the pipeline database provided by the local government. We have selected five locations to perform field measurements as shown in Figure 8, and specific pipe layouts in these locations are given by Figure 9. The leaks in water pipes occur mostly at the joint to connect pipes and the branch to distribute water to small pipes, and these leak points can be identified by checking manholes and the entrance of household pipes. Thus, we have found the actual leak point described as red spots in Figure 9 through the inspection of possible leak points as well as the leak detection based on the time difference estimation. The sound velocity in Figure 9 was determined by querying the database provided by [35] using the information about the material and diameter of the water supply pipe. Since two adjacent sensors are selected to log vibration signals for each field trial, there are nine kinds of test cases as shown in Table 3. For notational convenience, we use the test case names in Table 3 in Section 4 and Section 5.

### 5.2. Performance Evaluation

**Figure 10:**
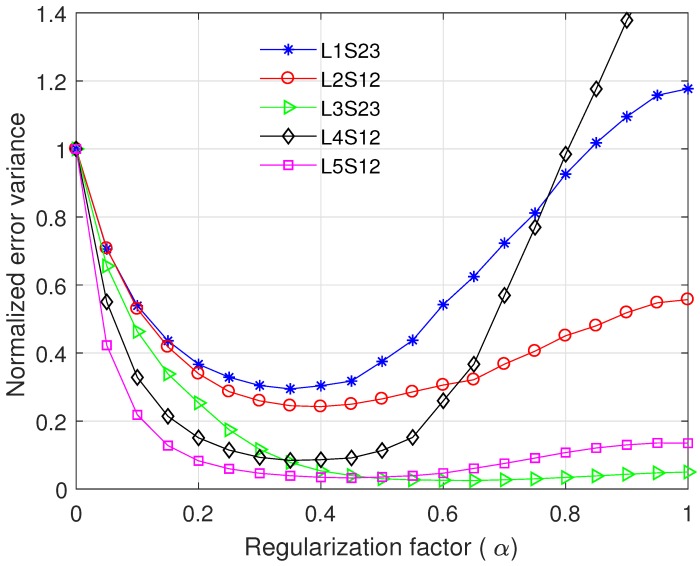
Normalised variance of time difference error as a function of α, when measured coherence values were used, N=4096, M=25, and $\sigma_{e}^{2}=0.02$.

In a practical system, the real coherence γ(k) is not available. Instead, γ(k) is estimated using the received signals $x_{1}\left(n\right)$ and $x_{2}\left(n\right)$ through multiple trials of measurement. Using γ(k) measured at five test locations, we compare the normalised variance of time difference error as a function of α as shown in Figure 10. The coherence function γ(k) was obtained by averaging three separate measurement data for each test case. We used N=4096 and M=25, and $\sigma_{e}^{2}$ was set to 0.02. For varying α, the normalised error variance presents huge variations depending on a test place because of the change of γ(k) and some interference by the external environment; however, the normalised error variance changes slightly when 0.30≤α≤0.55 having the minimum value between 0.35 and 0.60 as shown in Table 4. Based on the analysis in Figure 10, α was set to 0.45 for the proposed prefilter.

**Figure 11:**
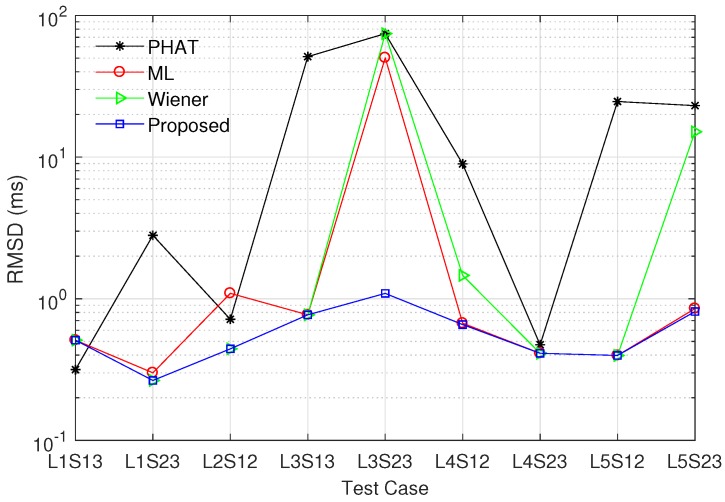
RMSD of various prefilters in field measurements, when α=0.45, N=4096, and M=25.

**Figure 12:**
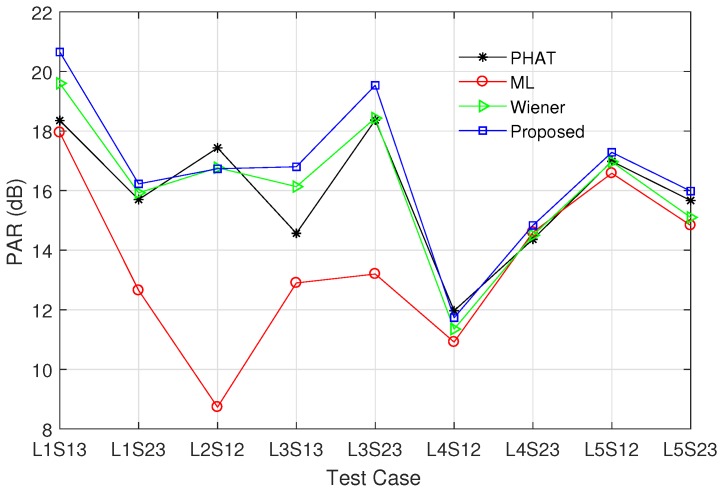
PAR of various prefilters in field measurements, when α=0.45, N=4096, and M=25.

As mentioned in Section 5.1, the field measurement was carried out for nine test cases in five locations. The field measurement was performed three times for each test case. We used N=4096 and M=25 for estimating the PSDs $S_{x_{1}}\left(k\right)$ and $S_{x_{2}}\left(k\right)$, and the CSD $S_{x_{1}x_{2}}\left(k\right)$; and α was set to 0.45 according to the analysis in Section 4.1. Based on the measurement data, Figure 11 and Figure 12 present the RMSD and the PAR of various prefilters, respectively. The proposed modified ML prefilter achieves the smallest RMSD value in all test cases except L1S13 in which the PHAT prefilter has slightly less RMSD than the proposed method. Note that, in the test case L1S13, the RMSD values of all prefilters are less than 0.6 ms, i.e., the proposed method achieves proper estimation accuracy for finding the leak location. The RMSD of the proposed method is less than 1.1 ms for entire test cases. In Figure 12, the proposed prefilter obtains the highest PAR in all test cases except L2S12 in which the PAR value of the PHAT prefilter is 0.7 dB greater than that of the proposed scheme. The PHAT and Wiener prefilters have similar PAR values; however, the RMSD of the PHAT prefilter is higher than that of the Wiener method as shown in Figure 11. In addition, while the ML and Wiener prefilters have similar RMSD values except L5S23, the ML method presents much lower PAR values than the Wiener prefilter. Thus, the Wiener prefilter is better than the PHAT and ML methods. Overall, the proposed method outperforms the conventional prefilters because it achieves the minimum RMSD value among all prefilters while maximising the PAR. In other words, when the distance between sensors are identical, the proposed prefilter has higher leak detection probability than the conventional methods. In addition, under a fixed RMSD requirement, the distance between sensors can be longer in the proposed prefilter than those in the conventional schemes, and thus the sensor deployment cost can be reduced.

## 6. Conclusions

This paper proposed a new time difference estimation method for leakage detection based on the modified ML prefilter with the regularisation factor. Through analysis and numerical simulations, we have found the optimal regularisation factor and compared the performance of the proposed prefilter with that of conventional prefilters. The proposed leak detection method was verified by field measurements using a practical leak detection system, and it was shown that the proposed method outperforms the conventional techniques in terms of RMSD and PAR. The proposed modified ML prefilter can be applied to correlation-based leak detection systems utilizing the STFT and the wavelet transform. Furthermore, the proposed method can contribute to developing an automatic leakage management solution that collects leakage data, alarms about the risk of leaks, and informs about the specific leak locations.

## Figures and Tables

**Table 1:** Sound velocity according to the pipe material and diameter [35].

Material	Diameter (mm)	Velocity (m/s)
	40	565
Polyvinyl Chloride (PVC)	80	540
	150	530
	150	1220
Cast-Iron	250	1160
	350	1120
	25	1375
	40	1350
Steel	60	1330
	90	1286
	150	1200
	250	1150

**Table 2:** Conventional prefilters for the generalised cross-correlation method [18,19,20].

Prefilter Type	Weight Function
Roth	$\frac{1}{S_{x_{1}}\left(k\right)}$
Smoothed coherence transform (SCOT)	$\frac{1}{\sqrt{S_{x_{1}}\left(k\right)S_{x_{2}}\left(k\right)}}$
Phase transform (PHAT)	$\frac{1}{|S_{x_{1}x_{2}}\left(k\right)|}$
Eckart	$\frac{|S_{x_{1}x_{2}}\left(k\right)|}{S_{w_{1}}\left(k\right)S_{w_{2}}\left(k\right)}$
Wiener	${\left|\gamma \left(k\right)\right|}^{2}$
Maximum-likelihood (ML)	$\frac{{\left|\gamma \left(k\right)\right|}^{2}}{1-{\left|\gamma \left(k\right)\right|}^{2}}\frac{1}{|S_{x_{1}x_{2}}\left(k\right)|}$

**Table 3:** Test cases in the field measurement.

Test Case	L1S13	L1S23	L2S12	L3S13	L3S23	L4S12	L4S23	L5S12	L5S23
Location	1	1	2	3	3	4	4	5	5
Sensor Pair	S1, S3	S2, S3	S1, S2	S1, S3	S2, S3	S1, S2	S2, S3	S1, S2	S2, S3

**Table 4:** Optimal regularisation factor minimising the time difference error variance in field measurements.

Test Case	L1S23	L2S12	L3S23	L4S12	L5S12
Optimal α	0.35	0.40	0.60	0.35	0.45

## References

[1] United States EPA Office of Water Control and Mitigation of Drinking Water Losses in Distribution Systems. https://nepis.epa.gov/.

[2] BDEW German Association of Energy and Water Industries VEWA Survey—Comparison of European Water and Wastewater Prices. https://www.bdew.de/.

[3] Ministry of Environment, Republic of Korea 2015 Statistics of Waterworks. http://me.go.kr/.

[4] Puust R., Kapelan Z., Savic D.A., Koppel T. (2010). A Review of Methods for Leakage Management in Pipe Networks. Urban Water J..

[5] Martini A., Troncossi M., Rivola A. (2015). Automatic Leak Detection in Buried Plastic Pipes of Water Supply Networks by Means of Vibration Measurements. Shock Vib..

[6] Liston D.A., Liston J.D. (1992). Leak Detection Techniques. J. N. Engl. Water Works Assoc..

[7] Hunaidi O. (2000). Detecting leaks in water-distribution pipes. Constr. Technol. Updates.

[8] Hunaidi O., Chu W., Wang A., Guan W. (2000). Detecting leaks in plastic pipes. J. Am. Water Works Assoc..

[9] Mounce S.R., Boxall J.B., Machell J. (2010). Development and Verification of an Online Artificial Intelligence System for Detection of Bursts and Other Abnormal Flows. J. Water Resour. Plan. Manag..

[10] Furthermoreersen J.H., Powell R.S. (2000). Implicit State-Estimation Technique for Water Network Monitoring. Urban Water.

[11] Poulakis Z., Valougeorgis D., Papadimitriou C. (2003). Leakage Detection in Water Pipe Networks Using a Bayesian Probabilistic Framework. Probab. Eng. Mech..

[12] Goulet J.A., Coutu S., Smith I.F. (2013). Model Falsification Diagnosis and Sensor Placement for Leak Detection in Pressurized Pipe Networks. Adv. Eng. Inf..

[13] Moser G., Paal S.G., Smith I.F. (2015). Performance Comparison of Reduced Models for Leak Detection in Water Distribution Networks. Adv. Eng. Inf..

[14] Ayala–Cabrera D., Herrera M., Izquierdo J., Ocaña–Levario S.J., Pérez–García R. (2013). GPR-Based Water Leak Models in Water Distribution Systems. Sensors.

[15] Cataldo A., Cannazza G., Benedetto E.D., Giaquinto N. (2012). A New Method for Detecting Leaks in Underground Water Pipelines. IEEE Sens. J..

[16] Giaquinto N., D’Aucelli G.M., Benedetto E.D., Cannazza G., Cataldo A., Piuzzi E., Masciullo A. (2016). Criteria for Automated Estimation of Time of Flight in TDR Analysis. IEEE Trans. Instrum. Meas..

[17] Hunaidi O., Wang A., Bracken M. Acoustic Methods for Locating Leaks in Municipal Water Pipe Networks. Proceedings of the International Conference Water Demand Management.

[18] Knapp C., Carter G. (1976). The Generalized Correlation Method for Estimation of Time Delay. IEEE Trans. Acoust. Speech Signal Process..

[19] Hero A., Schwartz S. (1985). A New Generalized Cross Correlator. IEEE Trans. Acoust. Speech Signal Process..

[20] Carter G.C. (1987). Coherence and Time Delay Estimation. Proc. IEEE.

[21] Gao Y., Brennan M., Joseph P. (2006). A Comparison of Time Delay Estimators for the Detection of Leak Noise Signals in Plastic Water Distribution Pipes. J. Sound Vib..

[22] Lay-Ekuakille A., Vendramin G., Trotta A., Vanderbemden P. STFT-Based Spectral Analysis of Urban Waterworks Leakage Detection. Proceedings of the XIX IMEKO World Congress Fundamental and Applied Metrology.

[23] Ge C., Yang H., Ye H., Wang G. (2009). A Fast Leak Locating Method Based on Wavelet Transform. Tsinghua Sci. Technol..

[24] Zhang L., Wu Y., Guo L., Cai P. (2013). Design and Implementation of Leak Acoustic Signal Correlator for Water Pipelines. Inf. Technol. J..

[25] Yang J., Wen Y., Li P. Information Processing for Leak Detection on Underground Water Supply Pipelines. Proceedings of the 3rd International Workshop on Advanced Computational Intelligence.

[26] Kadri A., Yaacoub E., Mushtaha M. Empirical Evaluation of Acoustical Signals for Leakage Detection in Underground Plastic Pipes. Proceedings of the 17th IEEE Mediterranean Electrotechnical Conference.

[27] Ahadi M., Bakhtiar M.S. (2010). Leak Detection in Water-Filled Plastic Pipes Through the Application of Tuned Wavelet Transforms to Acoustic Emission Signals. Appl. Acoust..

[28] Rashid S., Qaisar S., Saeed H., Felemban E. (2014). A Method for Distributed Pipeline Burst and Leakage Detection in Wireless Sensor Networks Using Transform Analysis. Int. J. Distrib. Sens. Netw..

[29] Bentoumi M., Chikouche D., Mezache A., Bakhti H. (2017). Wavelet DT Method for Water Leak-Detection Using a Vibration Sensor: An Experimental Analysis. IET Signal Process..

[30] Lay-Ekuakille A., Vendramin G., Trotta A. (2009). Robust Spectral Leak Detection of Complex Pipelines Using Filter Diagonalization Method. IEEE Sens. J..

[31] Almazyad A.S., Seddiq Y.M., Alotaibi A.M., Al-Nasheri A.Y., BenSaleh M.S., Obeid A.M., Qasim S.M. (2014). A Proposed Scalable Design and Simulation of Wireless Sensor Network-Based Long-Distance Water Pipeline Leakage Monitoring System. Sensors.

[32] Shi Y., Zhang C., Li R., Cai M., Jia G. (2015). Theory and Application of Magnetic Flux Leakage Pipeline Detection. Sensors.

[33] Muggleton J.M., Brennan M.J., Pinnington R.J. (2002). Wavenumber Prediction of Waves in Buried Pipes for Water Leak Detection. J. Sound Vib..

[34] Jackets T. (2014). The Newton-Laplace Equation and Speed of Sound. https://www.thermaxxjackets.com/newton-laplace-equation-sound-velocity/.

[35] Yoon D.J., Lee Y., Kim Y., Kim C., Jung J.C., Kim S.M., Lee J., Jeon H., Moon C., Kim E.C. (2004). Development of Leak Detection System for Waterworks Using Elastic Wave.

[36] Carter G.C. (1976). Time Delay Estimation. Ph.D. Thesis.

[37] Hayes M.H. (1996). Statistical Digital Signal Processing and Modeling.

